# Development and validation of a Score for Preoperative Prediction of Obstructive Sleep Apnea (SPOSA) and its perioperative outcomes

**DOI:** 10.1186/s12871-017-0361-z

**Published:** 2017-05-30

**Authors:** Christina H. Shin, Stephanie D. Grabitz, Fanny P. Timm, Noomi Mueller, Khushi Chhangani, Karim Ladha, Scott Devine, Tobias Kurth, Matthias Eikermann

**Affiliations:** 1 Department of Anesthesia, Critical Care, and Pain Medicine, Massachusetts General Hospital, 55 Fruit Street, Boston, Massachusetts USA; 2 Harvard Medical School, Boston, Massachusetts USA; 3 Department of Anesthesia, Toronto General Hospital and University of Toronto, Toronto, Canada; 4 Center for Observational and Real-World Effectiveness US Outcomes Research, Merck & Co., Inc, Boston, Massachusetts USA; 5 Institute of Public Health, Charité Universitätsmedizin Berlin, Berlin, Germany; 6 Brigham and Women’s Hospital, Boston, Massachusetts USA; 7 Universitaet Duisburg-Essen, Essen, Germany

**Keywords:** Perioperative obstructive sleep apnea, Prediction, Outcomes, Postoperative respiratory complications, In-hospital mortality

## Abstract

**Background:**

Postoperative respiratory complications (PRCs) are associated with significant morbidity, mortality, and hospital costs. Obstructive sleep apnea (OSA), often undiagnosed in the surgical population, may be a contributing factor. Thus, we aimed to develop and validate a score for preoperative prediction of OSA (SPOSA) based on data available in electronic medical records preoperatively.

**Methods:**

OSA was defined as the occurrence of an OSA diagnostic code preceded by a polysomnography procedure. *A priori* defined variables were analyzed by multivariable logistic regression analysis to develop our score. Score validity was assessed by investigating the score’s ability to predict non-invasive ventilation. We then assessed the effect of high OSA risk, as defined by SPOSA, on PRCs within seven postoperative days and in-hospital mortality.

**Results:**

A total of 108,781 surgical patients at Partners HealthCare hospitals (2007–2014) were studied. Predictors of OSA included BMI >25 kg*m^−2^ and comorbidities, including pulmonary hypertension, hypertension, and diabetes. The score yielded an area under the curve of 0.82. Non-invasive ventilation was significantly associated with high OSA risk (OR 1.44, 95% CI 1.22–1.69). Using a dichotomized endpoint, 26,968 (24.8%) patients were identified as high risk for OSA and 7.9% of these patients experienced PRCs. OSA risk was significantly associated with PRCs (OR 1.30, 95% CI 1.19–1.43).

**Conclusion:**

SPOSA identifies patients at high risk for OSA using electronic medical record-derived data. High risk of OSA is associated with the occurrence of PRCs.

**Electronic supplementary material:**

The online version of this article (doi:10.1186/s12871-017-0361-z) contains supplementary material, which is available to authorized users.

## Background

Obstructive sleep apnea (OSA), the most common type of sleep-disordered breathing, is characterized by recurrent partial or complete collapse of the upper airway during sleep. It is associated with significant immediate and long-term morbidity, including fragmented sleep, impaired daytime functioning, and reduced quality of life [1, 2]. OSA has also been associated with other medical conditions including hypertension [3], stroke [4], heart failure [5, 6], type 2 diabetes [7], obesity [8], and metabolic syndrome [9, 10].

OSA is a highly prevalent disease, affecting approximately 9 to 24% of the general population [11, 12]. However, these numbers may underestimate the true prevalence of the disease, as studies have shown that a significant proportion of OSA patients are undiagnosed [13–16].

Surgical patients with OSA are particularly vulnerable to perioperative morbidity [17], including postoperative respiratory complications (PRCs), as anesthesia and surgery affect the collapsibility of the upper airway as well as respiratory drive [18, 19]. The American Society of Anesthesiologists (ASA) has recently updated a set of practice guidelines for providers regarding the importance of preoperative screening for OSA through comprehensive review of medical records for history of comorbidities as well as any prior sleep studies, interview with patient and/or family, and physical examination [20]. To date, several prediction scores and questionnaires have been constructed. Those that have been validated in the perioperative period include the Perioperative Sleep Apnea Prediction Score (P-SAP) [21] and the STOP-Bang score [22]. Anesthesiologists have also used scores, such as the Mallampati Score, ASA Checklist, and the DES-OSA score [23] to assess difficulty of intubation as related to a narrow upper airway [24]. However, like P-SAP and STOP-Bang, these scores rely on a clinical exam and there is inconsistency in reported sensitivity and specificity of the Mallampati score as a predictor of OSA [25]. The currently available scores require data from an airway exam not routinely available from clinical databases, such as Mallampati class or thyromental distance. As a result, there is an emphasis on patient awareness and physician suspicion, both of which may fail to detect OSA with high sensitivity and specificity [26]. Often, some surgical patients do not see an anesthesiologist prior to the day of surgery and instead are screened preoperatively via phone interview in order to generate or update the electronic medical record. Given the strong associations between OSA and various comorbid diseases [27], it may be fruitful to utilize readily available data on demographics and comorbidities to make predictions regarding OSA risk.

The objectives of this study were to develop a prediction score based on patient data available in hospital-based electronic medical records, utilize the prediction score to characterize the impact of high OSA risk on PRCs, and assess whether or not intraoperative pharmacologic agents affect the association between high OSA risk and PRCs. We hypothesize that patients at high risk of OSA will also have higher risk of adverse postoperative outcomes.

## Methods

This study is an analysis of prospectively collected data on file using hospital-based electronic patient data at Massachusetts General Hospital, a tertiary care facility and teaching hospital of Harvard Medical School, as well as two community hospitals affiliated with Partners HealthCare in Massachusetts, United States of America. The protocol for this study has been previously peer-reviewed and published [28]. This project received approval from the Partners Institutional Review Board (Protocol #2014P000218).

As previously used for studies by our group, data from two clinical databases and one administrative database were retrieved and combined to provide de-identified pre- and postoperative information: the Research Patient Data Registry, the Anesthesia Information Management System, and Enterprise Performance Systems Inc [29–31]. The Research Patient Data Registry contains demographic and billing data regarding patient comorbidities and postoperative outcome and survival. The Anesthesia Information Management System contains physiological data from patient monitors as well as documentation of important surgery and anesthesia-related events, including adverse events, perioperative procedures, and drug and fluid therapy. The Enterprise Performance Systems Inc (EPSi) is a performance improvement and financial planning system containing data on admission and discharge statistics. Patient data from these databases are linked through unique patient identifiers and the variables utilized for our prediction model were abstracted to form one database. The present database spans from January 2007 to August 2014 and includes more than 145,000 surgical cases.

### Subject selection

We included all surgical patients aged 18 years or older who underwent general anesthesia and received endotracheal intubation or airway management by supraglottic airway device at our institution between January 2007 and August 2014 and who had removal of all airway management devices within the operating room after the procedure. Patients who underwent surgery in the 4 weeks prior to the study case were excluded. Finally, all patients with an American Society of Anesthesiologists physical classification (ASA) score of 6 (brain-dead patients undergoing organ procurement) were excluded from the study.

### Prediction model reference standard

The reference standard for the prediction model was defined as patients with an International Statistical Classification of Diseases and Related Health Problems, ninth revision (ICD-9) OSA diagnosis following the appearance of a polysomnography procedural (CPT, Current Procedural Terminology) code in our medical databases. From this specific sequence of events, we inferred that these patients had their clinically suspected OSA diagnosis confirmed by polysomnography. Our OSA endpoint was confirmed by a blind review of 100 total charts: 50 charts each were randomly selected from those cases positive for the OSA endpoint and those cases negative for the OSA endpoint. If available, sleep study results were reviewed as well as preoperative evaluation reports by anesthesiology providers and consultation notes. Diagnoses of “obstructive sleep apnea” and/or evidence of active use of “positive airway pressure” devices at home were considered positive for OSA.

### Set of predictor variables analyzed

A number of variables have been found to be associated with an increased prevalence of OSA and are currently used for different prediction tools for OSA in surgical patients. From our clinical databases, we included the following variables in our prediction score: age, BMI, gender, ASA score, and medical comorbidities using ICD-9 diagnostic codes. An additional document lists the ICD-9 diagnostic codes used to identify patient comorbidities in more detail [see Additional file 1]. All covariates included in the prediction model were present within 1 year prior to surgery date.

### Prediction model

Continuous, normally distributed variables were expressed as mean ± standard deviation (SD), while ordinal as median [interquartile range, (IQR)] and categorical variables as frequency (percentages) if not otherwise specified. Out of the *a priori* defined aforementioned group of predictor variables, we identified those variables that met an entry criteria of *p* = 0.01 using a multivariable logistic regression analysis with a forward selection procedure. The Hosmer-Lemeshow test was used to determine the goodness of fit of the final prediction model, with a *p*-value ≥ 0.05 indicative of no significant difference between the observed and expected outcome. The beta coefficient of each significant predictor was divided by the smallest coefficient and the results were then rounded to the nearest whole number to define the score point value [31]. The discriminative ability of the score for OSA was assessed using the c-statistic, which is equivalent to the area under the receiver operating characteristic curve (AUC) [32]. A cut-point was identified to optimize the sensitivity and specificity of the score for the OSA endpoint using the Youden Index [33].

### Prediction score validation

The prediction score, called the score for preoperative prediction of obstructive sleep apnea (SPOSA), was internally validated using ten-fold cross-validation approach and the root mean square error values were averaged across all estimations of the model. In this procedure, the study population was randomly separated into ten equal sized samples and of these, a single sample was reserved as the validation data for testing. We then developed a prediction score based on the other nine deciles of the data using the same modeling process as in the initial approach. The resulting score was applied in the validation sample which we previously left out in the modeling process and assessed the performance of the score in this subgroup. This cross-validation procedure was repeated ten times, with each of the ten samples used as the validation data. We aimed to test how well the final prediction model performed within each sample of the original population. In addition, we calculated the derived SPOSA for each surgical case and evaluated its predictive value in the dataset using a logistic regression model. The calculated c-statistic and the estimated probabilities for OSA were determined.

As an additional assessment for the clinical predictability of our score, we performed a multivariable logistic regression analysis to predict the outcome of non-invasive ventilation. In fact, recently published data suggest that the combination of polysomnography followed by receipt of a non-invasive ventilation device is highly specific for a true diagnosis of OSA [34]. We identified patients with a procedure code for non-invasive ventilation within seven days of surgery and investigated its association with OSA risk, as defined by SPOSA.

### Missing data

Twelve thousand six hundred forty five cases were excluded from the primary analysis due to missing data. In order to assess the impact of the excluded cases, we re-estimated beta-coefficients and odds ratios of the primary model using multiple imputation by chained equations. Variables with missing data were imputed using all covariates included in the prediction score. Five imputations were used. The model estimates were combined using variance estimates that combine imprecision both within and across imputations.

### Effect of OSA risk on perioperative outcomes

The primary postoperative outcome was a composite outcome, postoperative respiratory complications (PRCs), defined as the incidence of reintubation, pulmonary edema, pneumonia and/or respiratory failure within the first seven postoperative days. The primary outcome has been previously used and validated by chart review. Events were identified by ICD-9 diagnostic and CPT procedural codes obtained from the Research Patient Data Registry database [see Additional file 1]. Secondary outcomes included the aforementioned individual outcomes and in-hospital mortality.

With the primary exposure variable as OSA risk, defined as the dichotomized SPOSA score, we performed unadjusted logistic regression analyses and analyses adjusted for demographic variables, comorbidities, and procedure-related variables. Variables included in our adjusted model were the following: age, gender, BMI, ASA physical status classification, Charlson Comorbidity Index, duration of the surgical procedure, admission type, emergency status, duration of hypotension, procedure relative value units, volume of intraoperative fluids, dose of anesthesia (median dose of anesthetic agents corrected for age), opioids (calculated as total morphine equivalent dose), vasopressors, sedatives, neuromuscular blocking agents, neostigmine use, units of packed red blood cell transfusion, and median values for plateau and peep pressures. Variables were selected based on *a priori* knowledge about association patterns between covariates, OSA, and PRCs.

Finally, to evaluate the potential effect modification by intraoperative neuromuscular blockade, neostigmine, opioid, anesthetic, and sedative use on PRCs, we investigated the interaction effects between OSA risk and the intraoperative pharmacologic agent. Interaction terms consisted of two categorical variables, one of which was the dichotomized OSA risk classification based on the SPOSA cut-point. Neuromuscular blocking agents (NMBA) and neostigmine were classified as binary variables with respect to the use of these agents. For propofol, inhalational anesthetic (quantified as age-adjusted MAC), and morphine, we calculated the median dose within our population and created a binary variable based on high versus low dose of the respective agent. Confounder control was consistent with our main analyses. Results are presented as an unadjusted and multivariable-adjusted odds ratio (OR) with 95% confidence intervals (95% CI).

Statistical analyses were conducted by using the software STATA (Version 13.1, StataCorp, College Station, TX) and a two-sided *p*-value of <0.05 was considered statistically significant.

## Results

### Study cohort

**Fig. 1:**
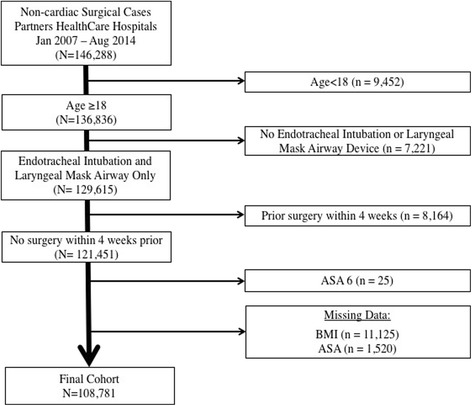
Study flow chart

A total of 146,288 surgical cases were identified. Of those a total of 37,264 cases were excluded because they either had missing values for covariates, received their care predominantly outside the main Massachusetts General Hospital, age was <18 years at the time of surgery, or did not undergo endotracheal intubation or placement of supraglottic airway device. In addition, patients with a surgical procedure within four weeks prior to the study case were excluded and only the first procedure remained in the cohort. The study flow is summarized in Fig. 1.

### Obstructive sleep apnea and patient characteristics

The modeling cohort is described in Table 1. Within the entire cohort, patients were on average 54 ± 16 years old and 56% were female. A total of 2,264 patients met our criteria for OSA based on a combination of an OSA diagnostic code preceded by occurrence of a polysomnography procedure code. Review of 100 randomly selected cases yielded a positive predictive value of 86% and a negative predictive value of 96% based on evidence of either AHI > 5 in polysomnography reports, active use of continuous positive airway pressure at home, or confirmation of OSA diagnosis in preoperative evaluation notes.

**Table 1:** Characteristics of study population

Variables	OSA patients (*n* = 2,264)	Non-OSA patients (*n* = 106,517)	All patients (*n* = 108,781)
Demographics			
Age (yrs), mean (SD)	54.9 (13.8)	54.4 (16.5)	54.4 (16.4)
Gender			
Male	1,143 (50.5%)	44,178 (44.3%)	48,321 (44.4%)
Female	1,121 (49.5%)	59,339 (55.7%)	60,460 (55.6%)
BMI (kg.m^−2^), mean (SD)	36.3 (9.4)	28.4 (7.0)	28.6 (7.1)
ASA status, median (IQR)	2 (2-3)	2 (2-3)	2 (2-3)
1	25 (1.1%)	10,386 (9.8%)	10,411 (9.8%)
2	1,147 (50.7%)	64,328 (60.4%)	65,475 (61.5%)
3	1,051 (46.4%)	30,243 (28.4%)	31,294 (28.4%)
4	40 (2.0%)	1,533 (1.4%)	1,573 (1.5%)
5	1 (0.04%)	27 (0.03%)	28 (0.03%)
Comorbidities			
Acute Ischemic Stroke	61 (2.7%)	1,714 (1.6%)	1,775 (1.6%)
Arterial Hypertension	1,646 (72.7%)	44,921 (42.2%)	46,567 (42.8%)
Atrial Fibrillation	306 (13.5%)	7,025 (6.6%)	7,331 (6.7%)
Cerebrovascular Disease	205 (9.0%)	8,224 (7.7%)	8,429 (7.7%)
Chronic Pulmonary Disease	664 (29.3%)	12,680 (11.9%)	13,344 (12.3%)
Congestive Heart Failure	403 (17.8%)	7,298 (6.9)	7,701 (7.1%)
Coronary Artery Disease	336 (14.8%)	7,221 (6.8%)	7,557 (6.9%)
Dementia	16 (0.7%)	551 (0.5%)	567 (0.5%)
Diabetes Mellitus	743 (32.8%)	12,855 (12.1%)	13,598 (12.5%)
Dyslipidemia	1,438 (63.5%)	34,349 (32.2%)	35,787 (32.9%)
Hemi/Paraplegia	76 (3.4%)	2,204 (2.1%)	2,280 (2.1%)
Liver Disease	608 (26.9%)	10,091 (9.5%)	10,699 (9.8%)
Myocardial Infarction	46 (2.0%)	1,259 (1.2%)	1,305 (1.2%)
Peptic Ulcer Disease	35 (1.5%)	673 (0.6%)	708 (0.7%)
Peripheral Vascular Disease	262 (11.6%)	8,169 (7.7%)	8,431 (7.8%)
Pulmonary Hypertension	165 (7.3%)	1,630 (1.5%)	1,795 (1.7%)

All values stated as number of patients (%), unless otherwise stated

*OSA* obstructive sleep apnea, *BMI* body mass index, *ASA* American Society of Anesthesiologists physical classification score

### Preoperative predictors for obstructive sleep apnea

**Fig. 2:**
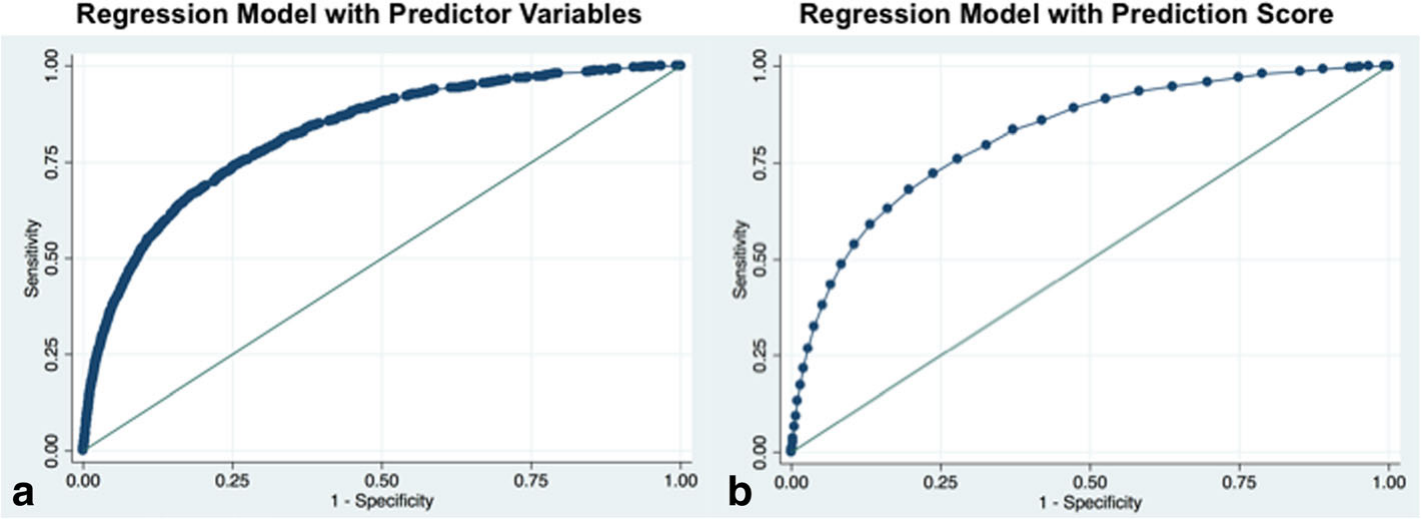
Receiver operating characteristic (ROC) curve for prediction of obstructive sleep apnea. **a** ROC curve was performed for the logistic regression model derived from significant independent predictors (AUC 0.8218). **b** A second ROC curve was fitted based on the composite prediction score derived from our prediction model (AUC 0.8211)

Based on the results of an unconditional multivariable logistic regression model with forward stepwise selection procedure, significant predictors included BMI > 25, ASA 2 to 4, age 18 to 70, and the following comorbidities: dyslipidemia, chronic pulmonary disease, liver disease, hypertension, congestive heart failure, pulmonary hypertension, atrial fibrillation, diabetes, coronary artery disease, and hemiplegia/paraplegia (Table 2). The final model yielded a c-statistic of 0.8211 (Fig. 2). The Hosmer-Lemeshow test demonstrated a well-calibrated model (*p* = 0.35).

**Table 2:** Prediction model for Obstructive Sleep Apnea

Predictor	Odds Ratio	*p*-value	95% Confidence Interval	Score Value
Male Gender	1.24	<0.001	1.14–1.36	1
BMI (kg.m^−2^)				
25 to <30	2.13	<0.001	1.78–2.55	4
30 to <35	4.04	<0.001	3.39–4.81	8
35+	8.50	<0.001	7.20–10.05	12
Age (yr)				
18–50	3.56	<0.001	2.68–4.71	7
50–70	2.35	<0.001	1.80–3.08	5
70–80	1.55	0.003	1.16–2.06	2
ASA				
2	3.28	<0.001	2.21–4.87	6
3	3.55	<0.001	2.37–5.32	7
4	2.16	0.004	1.28–3.67	4
Arterial Hypertension	1.67	<0.001	1.49–1.86	3
Atrial Fibrillation	1.40	<0.001	1.21–1.61	2
Chronic Pulmonary Disease	1.84	<0.001	1.66–2.05	3
Congestive Heart Failure	1.35	<0.001	1.18–1.55	2
Diabetes	1.24	0.001	1.12–1.37	1
Dyslipidemia	2.14	<0.001	1.93–2.37	4
Hemiplegia/Paraplegia	1.40	0.007	1.10–1.79	2
Liver Disease	1.97	<0.001	1.77–2.18	4
Pulmonary Hypertension	1.89	<0.001	1.55–2.31	3
Coronary Artery Disease	1.20	0.007	1.05–1.38	1

Odds ratios, *p*-values and 95% CI are presented for those predictor variables identified as the strongest independent predictors in a multivariable binary logistic regression model for obstructive sleep apnea. Predictors were assigned a rounded score point value in proportion to the lowest beta coefficient in the model

Based on the beta coefficients for the final model, point values were assigned to the predictors and are summarized in Table 2. The summed point values of the developed model ranged from 0 to 49 (median 19 (IQR 15-24)) points and were on average higher in patients who had ICD-9 codes for OSA versus who did not (median 29 (IQR 24-34) vs 19 (IQR 14-24), *p* < 0.001). We provide the predicted probabilities for each integer score [see Additional file 2]. The score led to a c-statistic of 0.82 (Fig. 2).

Using the Youden Index [33], we calculated a score value of 24 as cut-point for a dichotomization that optimizes the test performance of SPOSA for OSA. This cut-point identified 26,968 (24.8%) of the population as high risk for OSA with a score range of 25 to 49 (median 28 (IQR 26-31)). Patients at low risk for OSA had a range of 1 to 24 (median 17 (IQR 13-20)). The sensitivity and specificity of the SPOSA cut-point for our reference OSA standard were 72.3% (95% CI 70.4–74.1) and 76.2% (95% CI 76.0–76.5), respectively. The positive predictive value and negative predictive value were 6.1% (95% CI 5.8–6.4) and 99.2% (95% CI 99.2–99.3), respectively.

In order to classify a patient as having a low, moderate, and high risk for OSA, our study population was divided into three equal sized groups based on SPOSA. A total of 38,642 patients were considered low OSA risk with a range of 1 to 16 (median 13 (IQR 11-15)). 38,823 patients were identified as moderate OSA risk with a range of 17 to 23 (median 20 (18-22)). Finally, 31,316 patients were classified as high OSA risk with a range of 24 to 49 (median 27 IQR (25-31)).

### Validation of prediction score

Ten-fold cross-validation was performed for internal validation of our prediction model and yielded a root mean square error value of 0.0145 across ten iterations with a mean AUC of 0.8165.

We further sought to assess the validity of SPOSA by investigating its association with the incidence of non-invasive ventilation following surgery. This approach was based on the work of McIsaac and co-workers who reported that the combination of a polysomnography procedure code followed by receipt of a non-invasive ventilation device was highly specific for a true diagnosis of OSA (specificity of 98%) [34]. In our study, a total of 1,139 (4.2%) of patients at high risk of OSA (score >24) received non-invasive ventilation without subsequent reintubation within 7 days of surgery, compared with a total of 496 (0.6%) of patients at low risk of OSA (SPOSA ≤24). Multivariable logistic regression analyses, which controlled for a variety of potential intraoperative confounders, resulted in a significant association between high OSA risk (SPOSA >24) and the outcome of non-invasive ventilation (OR 1.44, 95% CI 1.22–1.69, *p* < 0.001).

### Missing data

In order to account for missing confounders, the primary regression analysis was repeated using multiple imputations by chained equations. The association of all previously identified independent predictors and OSA remained significant with similar odds ratios [see Additional file 3].

### Association between postoperative respiratory complications and OSA risk

A total of 5,894 (5.4%) patients experienced a PRC within 7 days of surgery. The breakdown of individual respiratory complications was as follows: 3,936 (3.6%) pulmonary edema, 1,459 (1.3%) pneumonia, 1,927 (1.8%) respiratory failure, and 410 (0.4%) reintubation. Increasing OSA risk, as quantified by SPOSA in tertiles, was significantly associated with higher odds of PRCs in an unadjusted model (Table 3). This association remained stable after adjustments (Table 3).

**Table 3:** Association of increasing Obstructive Sleep Apnea risk and Postoperative Respiratory Complications (PRC)

SPOSA Score	Frequency of PRC (%)	Unadjusted OR (95% CI), *p*-value	Adjusted OR (95% CI), *p*-value
Low: (1 to 16)	1,357 (3.5%)	1	1
Moderate: (17 to 23)	2,106 (5.4%)	1.58 (1.47–1.69), *p* < 0.001	1.14 (1.04–1.24), *p* = 0.004
High: (24 to 49)	2,431 (7.8%)	2.31 (2.15–2.48), *p* < 0.001	1.42 (1.25–1.59), *p* < 0.001

The results of unadjusted and adjusted multivariable logistic regression analyses are presented below as odds ratios (OR), 95% Confidence Interval (95% CI), *p*-value

A total of 2,126 patients (7.9%) of patients identified as high OSA risk (SPOSA > 24) experienced PRCs within 7 days following surgery, while a total of 3,768 (4.6%) of low OSA risk (SPOSA ≤ 24) patients were positive for this outcome (Table 4). In unadjusted and adjusted analyses, high OSA risk was significantly associated with PRCs (adjusted OR 1.30, 95% CI 1.19–1.43, *p* < 0.001; Table 4). Of the individual respiratory complications, this effect seemed to be driven largely by pulmonary edema (Table 4).

**Table 4:** Association between Obstructive Sleep Apnea (OSA) risk and postoperative outcomes

Outcome	High OSA Risk (SPOSA >24) N (%)	Low OSA Risk (SPOSA ≤ 24) N (%)	Unadjusted OR (95% CI), *p*-value	Adjusted OR (95% CI), *p*-value
Postoperative Respiratory Complications	2,126 (7.9%)	3,768 (4.6%)	1.77 (1.68–1.87), *p* < 0.001	1.30 (1.19–1.43), *p* < 0.001
Pulmonary Edema	1,519 (5.6%)	2,417 (3.0%)	1.96 (1.84–2.09), *p* < 0.001	1.48 (1.33–1.64), *p* < 0.001
Pneumonia	495 (1.8%)	964 (1.2%)	1.57 (1.41–1.75), *p* < 0.001	1.13 (0.96–1.33), *p* = 0.14
Respiratory Failure	626 (2.3%)	1,301 (1.6%)	1.47 (1.34–1.62), *p* < 0.001	0.96 (0.83–1.11), *p* = 0.61
Reintubation	125 (0.5%)	285 (0.3%)	1.33 (1.08–1.64), *p* = 0.008	0.89 (0.65–1.22), *p* = 0.48
In-hospital mortality	98 (0.4%)	277 (0.3%)	1.08 (0.85–1.35), *p* = 0.54	0.74 (0.53–1.03), *p* = 0.071

Results of unadjusted and adjusted multivariable logistic regression analyses presented as odds ratio (OR), 95% Confidence Interval (95% CI), *p*-value

In-hospital death occurred in 98 (0.4%) patients with a SPOSA > 24 and in 277 (0.3%) patients with a SPOSA ≤24. There was no significant association between in-hospital mortality and OSA risk (adjusted OR 0.74, 95% CI 0.53–1.03, *p* = 0.071; Table 4).

### Effect modification by intraoperative pharmacologic agents

We found no significant interaction effects between OSA risk and each of the following pharmacologic agents used intraoperatively: neostigmine, NMBA, inhalational anesthetic, morphine, and propofol (Table 5).

**Table 5:** Interaction Effects between Obstructive Sleep Apnea (OSA) Risk and Intraoperative Pharmacologic agents

Interaction Term	Association between PRC and OSA Risk (Adjusted OR, (95% CI), *p*-value)
OSA Risk * Morphine Dose	1.04 (0.92–1.18), *p* = 0.55
OSA Risk * Age Adjusted MAC	0.98 (0.86–1.11), *p* = 0.72
OSA Risk * Propofol Dose	1.00 (0.88–1.13), *p* = 0.97
OSA Risk * NMBA Use	0.96 (0.79–1.16), *p* = 0.65
OSA Risk * Neostigmine Use	0.94 (0.82–1.08), *p* = 0.36

The results of adjusted multivariable logistic regression analyses are presented below for the interaction term of OSA Risk and Intraoperative Pharmacologic Agent as odds ratio (OR), 95% Confidence Interval (95% CI), *p*-value

## Discussion

We have developed a novel prediction score for OSA using data available in electronic medical records alone. The SPOSA is based on demographic data and data on medical comorbidities identified as predictors for OSA. Our score yielded an AUC of 0.8211 and identified 24.8% of our population as high OSA risk based on a cut-point of 24, which was optimized based on sensitivity and specificity for the condition of OSA. Our score was validated internally using cross-validation and its clinical validity was further assessed by its prediction of non-invasive ventilation within 7 days after surgery.

The SPOSA is a weighted model containing comorbidity and demographic variables known to be associated with OSA, including: chronic pulmonary disease [35], congestive heart failure [5, 6], diabetes [7], dyslipidemia [36, 37], hypertension [3, 38], atrial fibrillation [39, 40], liver disease [41], coronary artery disease [42], and pulmonary hypertension [43, 44]. The association between hemiplegia/paraplegia and OSA may be related to the an association between OSA and acute ischemic stroke [4, 45]. Male gender, high BMI, and older age have also been shown to predict OSA and are included in our model [8, 9, 12, 46].

We identified a cut-point to optimize the test performance of the SPOSA, which led to a 72 and 76% sensitivity and specificity, respectively. We were uncertain as to whether false positives were equally as costly as false negatives. The true costs in healthcare can be analyzed using the endpoint, value of care – that is, patient’s outcome as a fraction of monetary costs. Patients with a false positive diagnosis of OSA may be subsequently exposed to unnecessary perioperative interventions. A false negative OSA test carries an even higher risk for the patient, given the propensity for developing PRCs, which is harmful for the patient and costly for the hospital. Further studies are needed to define the consequences of false positive and false negative screening results.

Previous scores have been developed to predict the likelihood of OSA in a surgical patient. These scores require information derived from a clinical exam, such as the Mallampati class or thyromental distance, in patients prior to their scheduled procedure. The STOP-Bang [22] and P-SAP scores [21] are well-known and validated scores and may be used to risk stratify patients for postoperative complications [47]. However, to the best of our knowledge, no score for OSA exists that has been developed from electronic medical records that does not contain information derived from direct patient encounters or physical examinations and is applicable to a surgical patient population. Further, the SPOSA is a risk stratification tool for postoperative complications while adjusting for procedure-related factors, including surgical complexity.

Patient information available in electronic medical records and health administrative databases confer the advantage of studying large populations and screening the individual preoperative patient in a relatively inexpensive and more accessible way. Current methods of detecting OSA rely on patient-reported symptoms and physician-led examinations. However, many surgical patients opt for a phone interview with a non-anesthesia provider instead of an in-person preoperative evaluation by an anesthesia provider and thus many patients who may be at risk for OSA are missed in the screening process due to lack of physical exam information. Still, during these phone interviews, information on patient demographics and comorbidities are updated in the electronic record and this readily available data may be used for important screening efforts. A recent study performed amongst non-surgical patients concluded that non-symptom medical history was superior over patient-reported symptoms [48]. Using a new machine learning method (Supersparse Linear Integer Model, SLIM), authors identified the following variables as predictive for OSA in a sleep-lab referred population: older age, high BMI, diabetes, hypertension, smoker, and male gender. Compared with symptom-based features, a model based on history alone demonstrated a significantly higher AUC (0.78 vs. 0.67, *p* < 0.0001) [48]. The findings of Ustun and co-workers support the rationale and findings of our study as we sought to create a prediction model based on data available in the medical record. Our data add to the findings of Ustun and co-workers that an instrument composed of an optimal combination of comorbidities and BMI reliably predicts OSA risk in a surgical cohort.

The performance of the SPOSA is comparable to scores that have been developed in clinic-based settings. For example, the c-statistic for STOP-Bang and P-SAP score are 0.65 [49] and 0.79 [21], respectively, for an AHI > 5 compared to a c-statistic of 0.82 of our score. Of note, the STOP-Bang and P-SAP score require biological measurements to be taken during an exam (neck circumference, thyromental distance). The good test performance of the SPOSA – an instrument that does not utilize biomarkers (physical exam or lab information) – is consistent with other examples in which scores that do not include biomarkers perform very similarly to scores that do include such information [50–52]. For example, in a cardiovascular risk prediction score with and without laboratory measurements, such as cholesterol or c-reactive protein, predictive measures have been very similar [50, 51], which is very important for applications in more general settings or in regions where laboratory data are not easily ascertainable.

The SPOSA substantially adds to other available scores and allows providers to identify patients at increased risk of OSA from existing preoperative data resources. Of note, in a study by McIsaac and colleagues, the authors demonstrate that OSA specific billing codes alone are insufficient to identify patients with OSA [34]. Therefore, in order to generate the SPOSA, we utilized a different approach: we use a combination of available data on OSA associated comorbidities and other known predictors such as BMI, age, and gender. Our data show that a substantially high proportion of our cohort (about one third) presents with high risk of OSA whereas only 2.1% carried the OSA specific billing codes.

### SPOSA is associated with adverse respiratory events

In our cohort of surgical patients, we also found that a high preoperative risk of OSA, as defined by SPOSA, was associated with increased odds of PRCs. Our results remained stable after accounting for patient comorbidities and perioperative factors, suggesting that the association between high OSA risk and PRCs is likely a consequence of OSA risk, rather than a consequence of OSA-associated comorbidities.

OSA has been established as a risk factor for adverse perioperative outcomes. The higher propensity of patients with high risk for OSA towards PRCs is most likely multifactorial and related to a pathological imbalance of upper airway dilation and collapse. We hypothesized that surgical patients at high risk for OSA are especially vulnerable to multiple perioperative insults, including the effects of sedatives, opioids, neuromuscular blocking agents, fluid resuscitation, and more. In order to evaluate the biological implications of high OSA risk, we performed our primary analysis and investigated the association between high OSA risk, as defined by SPOSA > 24, and incidence of PRCs as a composite outcome within seven postoperative days while controlling for several perioperative factors. We report a significant association between high preoperative OSA risk, as defined by increasing SPOSA values, and increased rate of PRCs in non-cardiac surgery patients. Of note, the primary driver of this association appeared to stem from a significant association between high preoperative OSA risk and pulmonary edema. High OSA risk did not appear to have a significant impact on the remaining components of our composite primary outcome: reintubation, respiratory failure, and pneumonia. Our findings are supported by other work in the literature, which have primarily associated a medical diagnosis of sleep-disordered breathing with cardiopulmonary complications [53, 54].

Emergent intubation or reintubation is a common respiratory endpoint studied in the context of OSA patients and many studies have demonstrated a significant association [17, 55]. However, our findings differ in that our increased rate of reintubation observed among patients with a high SPOSA score was, although increased, not statistically significant. The absence of a significant association between high OSA risk and reintubation may be in part due to the significantly increased rates of postoperative noninvasive ventilation among high OSA risk patients found in our study. While the authors of a meta-analysis of the association between OSA and postoperative outcomes did not specifically investigate noninvasive ventilation, they also found a non-significant association between reintubation and OSA patients, consistent with our findings and that of an updated meta-analysis [54, 56].

### Biological implications

We found that high preoperative risk of OSA increases the risk of developing respiratory complications within 7 days following surgery, a finding that is consistent with several studies in the literature. This association was stable across various methods of SPOSA categorization. The significant association between high OSA risk and PRCs may be in part driven by the high rate of negative pressure pulmonary edema in this vulnerable group of patients following surgery [57, 58]. Another potential driving force of pulmonary edema formation is the phenomenon of overnight rostral fluid shifts. Redolfi et al. measured leg fluid volumes and performed overnight polysomnography in healthy, non-obese men and found that overnight changes in lower extremity edema correlated significantly with AHI and neck circumference [59]. Perioperative fluid shifts should expose patients with high preoperative scores to an increased risk for perioperative airway obstruction, but rostral fluid shifts secondary to the positioning of patients during surgery and in the hospital may significantly contribute to adverse outcomes.

In contrast to a few studies [17, 55] which reported lower perioperative mortality among OSA patients, we did not find a significant association of in-hospital mortality among patients identified as high risk for OSA. One possible explanation for the difference in findings may lie in the populations studied. Nearly half of the NIS populations studied by Mokhlesi et al. were composed of patients receiving care at non-academic practices. In contrast, our study population is derived solely from a large academic institution and its two close affiliates in Boston, Massachusetts. In addition to differences in institutional practice, variations in hospital volumes may also drive the observed differences in in-hospital mortality [60]. Our data support the view that OSA is a clinically meaningful disorder in perioperative medicine.

### Strength and limitations

The SPOSA was derived from a large study cohort and a large number of patients with ICD-9 codes for OSA, allowing us to robustly develop a prediction score for OSA. Our database contains a variety of surgical procedure types and methods of anesthesia, thus increasing the generalizability of the study results and applicability of our prediction score model. In addition, the validity of our score has been assessed by its prognostic ability – that is, its ability to predict non-invasive ventilation early after surgery, an outcome that is known to occur more frequently amongst patients with OSA [61].

Several limitations have to be considered when interpreting our results. The SPOSA relies on the investigation of electronic patient data on file. Thus, our findings depend on the quality of the database, which is susceptible to measurement biases. There is potential for variability in the input of billing diagnoses and codes. This database has been used in previous studies [29–31] and demonstrated to have high specificity following verification of diagnostic codes. We have confirmed the accuracy of our unique combination of diagnostic and procedural codes in capturing patients with known OSA by medical record review. Nevertheless, it is possible that information is left out of some patients’ charts and consequently, our database of our composite outcomes and independent variables. While our work has been validated internally and has demonstrated to predict an outcome well associated with OSA, our score has not yet been validated in an external population or tested for its prediction of AHI, the gold standard for OSA diagnosis. Future studies by our group will be directed towards utilizing electronic medical record data to predict OSA as measured by AHI.

### Clinical implications

The SPOSA can be used as a preoperative screening instrument in patients prior to hospital admission, without the need of an in-person airway physical exam. Our findings of increased adverse outcomes among patients identified as high risk of OSA have important implications for members of the perioperative team. It is imperative that patients at greater surgical vulnerability be identified preoperatively. This tool may prompt providers to pursue further diagnostic evaluation prior to surgery as well as any planned treatment interventions, including an “OSA bundle” [62]. Given the increased risk of adverse respiratory outcomes among high risk OSA patients, perioperative providers should administer postoperative opioids cautiously [62] and utilize continuous positive airway pressure therapy in the PACU to mitigate postoperative opioid associated respiratory depression [63].

In addition, SPOSA may further be utilized by hospital administrators and clinicians to guide allocation of key resources, including staff and monitoring equipment, as patients identified as high risk of OSA are flagged for the perioperative OSA bundle.

## Additional files

Additional file 1: Table S1. Diagnostic (ICD-9) and Procedural (CPT) codes used to generate predictor and outcome variables. (DOCX 17 kb)

Additional file 2: Table S2. Predicted Probabilities for diagnosed Obstructive Sleep Apnea (OSA) at each integer of OSA risk prediction score. (DOCX 12 kb)

Additional file 3: Table S3. Accounting for missing data using multiple imputations by chained equations. (DOCX 14 kb)

## Abbreviations

AHI: Apnea hypopnea index
ASA: American Society of Anesthesiologists
AUC: Area under receiver operating curve
BMI: Body mass index
CI: Confidence interval
CPT: Current procedural terminology
ICD-9: International Statistical Classification of Disease and Related Health Problems, ninth revision
IQR: Interquartile range
OR: Odds ratio
OSA: Obstructive sleep apnea
PRCs: Postoperative respiratory complications
P-SAP: Perioperative sleep apnea prediction score
ROC: Receiver operating curve
SD: Standard deviation
SPOSA: Score for preoperative prediction of obstructive sleep apnea

## References

[1] Lacasse Y, Godbout C, Series F (2002). Health-related quality of life in obstructive sleep apnoea. Eur Respir J.

[2] Engleman HM, Douglas NJ (2004). Sleep. 4: Sleepiness, cognitive function, and quality of life in obstructive sleep apnoea/hypopnoea syndrome. Thorax.

[3] Logan AG, Perlikowski SM, Mente A, Tisler A, Tkacova R, Niroumand M (2001). High prevalence of unrecognized sleep apnoea in drug-resistant hypertension. J Hypertens.

[4] Lyons OD, Ryan CM (2015). Sleep apnea and stroke. Can J Cardiol.

[5] Naughton MT (2015). Respiratory sleep disorders in patients with congestive heart failure. J Thorac Dis.

[6] Stansbury RC, Strollo PJ (2015). Clinical manifestations of sleep apnea. J Thorac Dis.

[7] Kent BD, Grote L, Ryan S, Pépin J-L, Bonsignore MR, Tkacova R (2014). Diabetes mellitus prevalence and control in sleep-disordered breathing: the European Sleep Apnea Cohort (ESADA) study. Chest.

[8] Isono S (2012). Obesity and obstructive sleep apnoea: mechanisms for increased collapsibility of the passive pharyngeal airway. Respirology.

[9] Coughlin SR, Mawdsley L, Mugarza JA, Calverley PMA, Wilding JPH (2004). Obstructive sleep apnoea is independently associated with an increased prevalence of metabolic syndrome. Eur Heart J.

[10] Gruber A, Horwood F, Sithole J, Ali NJ, Idris I (2006). Obstructive sleep apnoea is independently associated with the metabolic syndrome but not insulin resistance state. Cardiovasc Diabetol.

[11] Memtsoudis SG, Besculides MC, Mazumdar M (2013). A rude awakening--the perioperative sleep apnea epidemic. N Engl J Med.

[12] Peppard PE, Young T, Barnet JH, Palta M, Hagen EW, Hla KM (2013). Increased prevalence of sleep-disordered breathing in adults. Am J Epidemiol.

[13] Young T, Evans L, Finn L, Palta M (1997). Estimation of the clinically diagnosed proportion of sleep apnea syndrome in middle-aged men and women. Sleep.

[14] Finkel KJ, Searleman AC, Tymkew H, Tanaka CY, Saager L, Safer-Zadeh E (2009). Prevalence of undiagnosed obstructive sleep apnea among adult surgical patients in an academic medical center. Sleep Med.

[15] Singh M, Liao P, Kobah S, Wijeysundera DN, Shapiro C, Chung F (2013). Proportion of surgical patients with undiagnosed obstructive sleep apnoea. Br J Anaesth.

[16] Wolfe RM, Pomerantz J, Miller DE, Weiss-Coleman R, Solomonides T (2016). Obstructive sleep apnea: preoperative screening and postoperative care. J Am Board Fam Med.

[17] Mokhlesi B, Hovda MD, Vekhter B, Arora VM, Chung F, Meltzer DO (2013). Sleep-disordered breathing and postoperative outcomes after elective surgery. Chest.

[18] Sasaki N, Meyer MJ, Eikermann M (2013). Postoperative respiratory muscle dysfunction: pathophysiology and preventive strategies. Anesthesiology.

[19] Fouladpour N, Jesudoss R, Bolden N, Shaman Z, Auckley D (2016). Perioperative complications in obstructive sleep apnea patients undergoing surgery: a review of the legal literature. Anesth Analg.

[20] American Society of Anesthesiologists Task Force on Perioperative Management of patients with obstructive sleep apnea (2014). Practice guidelines for the perioperative management of patients with obstructive sleep apnea: an updated report by the American Society of Anesthesiologists Task Force on Perioperative Management of patients with obstructive sleep apnea. Anesthesiology. The American Society of A. Anesthesiology.

[21] Ramachandran SK, Kheterpal S, Consens F, Shanks A, Doherty TM, Morris M (2010). Derivation and validation of a simple perioperative sleep apnea prediction score. Anesth Analg.

[22] Chung F, Yegneswaran B, Liao P, Chung SA, Vairavanathan S, Islam S (2008). STOP questionnaire: a tool to screen patients for obstructive sleep apnea. Anesthesiology.

[23] Deflandre E, Degey S, Brichant J-F, Poirrier R, Bonhomme V (2016). Development and validation of a morphologic obstructive sleep apnea prediction score: the DES-OSA score. Anesth Analg.

[24] Chung F, Yegneswaran B, Liao P, Chung SA, Vairavanathan S, Islam S (2008). Validation of the Berlin questionnaire and American Society of Anesthesiologists checklist as screening tools for obstructive sleep apnea in surgical patients. Anesthesiology.

[25] Abrishami A, Khajehdehi A, Chung F (2010). A systematic review of screening questionnaires for obstructive sleep apnea. Can J Anaesth.

[26] Skomro RP, Kryger MH (1999). Clinical presentations of obstructive sleep apnea syndrome. Prog Cardiovasc Dis.

[27] Eastwood PR, Malhotra A, Palmer LJ, Kezirian EJ, Horner RL, Ip MS (2010). Obstructive sleep apnoea: from pathogenesis to treatment: current controversies and future directions. Respirology.

[28] Shin CH, Zaremba S, Devine S, Nikolov M, Kurth T, Eikermann M (2016). Effects of obstructive sleep apnoea risk on postoperative respiratory complications: protocol for a hospital-based registry study. BMJ Open.

[29] McLean DJ, Diaz-Gil D, Farhan HN, Ladha KS, Kurth T, Eikermann M (2015). Dose-dependent association between intermediate-acting neuromuscular-blocking agents and postoperative respiratory complications. Anesthesiology.

[30] Ladha K, Vidal Melo MF, McLean DJ, Wanderer JP, Grabitz SD, Kurth T (2015). Intraoperative protective mechanical ventilation and risk of postoperative respiratory complications: hospital based registry study. BMJ.

[31] Brueckmann B, Villa-Uribe JL, Bateman BT, Grosse-Sundrup M, Hess DR, Schlett CL (2013). Development and validation of a score for prediction of postoperative respiratory complications. Anesthesiology.

[32] Hanley JA, McNeil BJ (1982). The meaning and use of the area under a receiver operating characteristic (ROC) curve. Radiology.

[33] Liu X (2012). Classification accuracy and cut point selection. Stat Med.

[34] McIsaac DI, Gershon A, Wijeysundera D, Bryson GL, Badner N, van Walraven C (2015). Identifying obstructive sleep apnea in administrative data: a study of diagnostic accuracy. Anesthesiology.

[35] Soler X, Gaio E, Powell FL, Ramsdell JW, Loredo JS, Malhotra A (2015). High prevalence of obstructive sleep apnea in patients with moderate to severe chronic obstructive pulmonary disease. Ann Am Thorac Soc.

[36] Xu H, Guan J, Yi H, Zou J, Meng L, Tang X (2016). Elevated low-density lipoprotein cholesterol is independently associated with obstructive sleep apnea: evidence from a large-scale cross-sectional study. Sleep Breath.

[37] Trzepizur W, Le Vaillant M, Meslier N, Pigeanne T, Masson P, Humeau MP (2013). Independent association between nocturnal intermittent hypoxemia and metabolic dyslipidemia. Chest.

[38] Shahar E, Whitney CW, Redline S, Lee ET, Newman AB, Nieto FJ (2001). Sleep-disordered breathing and cardiovascular disease: cross-sectional results of the Sleep Heart Health Study. Am J Respir Crit Care Med.

[39] Gami AS, Pressman G, Caples SM, Kanagala R, Gard JJ, Davison DE (2004). Association of atrial fibrillation and obstructive sleep apnea. Circulation.

[40] Qureshi WT, Nasir UB, Alqalyoobi S, O’Neal WT, Mawri S, Sabbagh S (2015). Meta-analysis of continuous positive airway pressure as a therapy of atrial fibrillation in obstructive sleep apnea. Am J Cardiol.

[41] Chou T-C, Liang W-M, Wang C-B, Wu T-N, Hang L-W (2015). Obstructive sleep apnea is associated with liver disease: a population-based cohort study. Sleep Med.

[42] Khayat R, Pleister A (2016). Consequences of obstructive sleep apnea: cardiovascular risk of obstructive sleep apnea and whether continuous positive airway pressure reduces that risk. Sleep Med Clin.

[43] Bady E, Achkar A, Pascal S, Orvoen-Frija E, Laaban JP (2000). Pulmonary arterial hypertension in patients with sleep apnoea syndrome. Thorax.

[44] Kholdani C, Fares WH, Mohsenin V (2015). Pulmonary hypertension in obstructive sleep apnea: is it clinically significant? A critical analysis of the association and pathophysiology. Pulm Circ.

[45] Johnson KG, Johnson DC (2010). Frequency of sleep apnea in stroke and TIA patients: a meta-analysis. J Clin Sleep Med.

[46] Young T, Palta M, Dempsey J, Skatrud J, Weber S, Badr S (1993). The occurrence of sleep-disordered breathing among middle-aged adults. N Engl J Med.

[47] Dimitrov L, Macavei V (2016). Can screening tools for obstructive sleep apnea predict postoperative complications? A systematic review of the literature. J Clin Sleep Med.

[48] Ustun B, Westover MB, Rudin C, Bianchi MT (2016). Clinical prediction models for sleep apnea: the importance of medical history over symptoms. J Clin Sleep Med.

[49] Chung F, Subramanyam R, Liao P, Sasaki E, Shapiro C, Sun Y (2012). High STOP-Bang score indicates a high probability of obstructive sleep apnoea. Br J Anaesth.

[50] Gaziano TA, Young CR, Fitzmaurice G, Atwood S, Gaziano JM (2008). Laboratory-based versus non-laboratory-based method for assessment of cardiovascular disease risk: the NHANES I Follow-up Study cohort. Lancet.

[51] D’Agostino RB, Vasan RS, Pencina MJ, Wolf PA, Cobain M, Massaro JM (2008). General cardiovascular risk profile for use in primary care: the Framingham Heart Study. Circulation.

[52] Stephan BCM, Tzourio C, Auriacombe S, Amieva H, Dufouil C, Alpérovitch A (2015). Usefulness of data from magnetic resonance imaging to improve prediction of dementia: population based cohort study. BMJ.

[53] Memtsoudis SG, Stundner O, Rasul R, Chiu YL, Sun X, Ramachandran SK (2014). The impact of sleep apnea on postoperative utilization of resources and adverse outcomes. Anesth Analg.

[54] Gaddam S, Gunukula SK, Mador MJ (2014). Post-operative outcomes in adult obstructive sleep apnea patients undergoing non-upper airway surgery: a systematic review and meta-analysis. Sleep Breath.

[55] Mokhlesi B, Hovda MD, Vekhter B, Arora VM, Chung F, Meltzer DO (2013). Sleep-disordered breathing and postoperative outcomes after bariatric surgery: analysis of the nationwide inpatient sample. Obes Surg.

[56] Kaw R, Chung F, Pasupuleti V, Mehta J, Gay PC, Hernandez AV (2012). Meta-analysis of the association between obstructive sleep apnoea and postoperative outcome. Br J Anaesth.

[57] Krodel DJ, Bittner EA, Abdulnour R, Brown R, Eikermann M (2010). Case scenario: acute postoperative negative pressure pulmonary edema. Anesthesiology.

[58] Krodel DJ, Bittner EA, Abdulnour R-EE, Brown RH, Eikermann M (2011). Negative pressure pulmonary edema following bronchospasm. Chest.

[59] Redolfi S, Yumino D, Ruttanaumpawan P, Yau B, Su M-C, Lam J (2009). Relationship between overnight rostral fluid shift and Obstructive Sleep Apnea in nonobese men. Am J Respir Crit Care Med.

[60] Pieper D, Mathes T, Neugebauer E, Eikermann M (2013). State of evidence on the relationship between high-volume hospitals and outcomes in surgery: a systematic review of systematic reviews. J Am Coll Surg.

[61] Lindenauer PK, Stefan MS, Johnson KG, Priya A, Pekow PS, Rothberg MB (2014). Prevalence, treatment, and outcomes associated with OSA among patients hospitalized with pneumonia. Chest.

[62] Zaremba S, Mojica JE, Eikermann M. Perioperative sleep apnea: a real problem or did we invent a new disease? F1000Res. 2016;5. 10.12688/f1000research.7218.1 PMC4797892 27006758

[63] Zaremba S, Shin CH, Hutter MM, Malviya SA, Grabitz SD, MacDonald T (2016). Continuous positive airway pressure mitigates opioid-induced worsening of sleep-disordered breathing early after bariatric surgery. Anesthesiology.

