# Supplementary material for: Development and validation of a Score for Preoperative Prediction of Obstructive Sleep Apnea (SPOSA) and its perioperative outcomes
Source: BMC Anesthesiol. 2017 May 30;17:71. doi: 10.1186/s12871-017-0361-z (PMC5450400; doi:10.1186/s12871-017-0361-z)
Supplement: Supplementary file 1 — Diagnostic (ICD-9) and Procedural (CPT) codes used to generate predictor and outcome variables. (DOCX 17 kb) [file 12871_2017_361_MOESM1_ESM.docx]

| **Table S1: Diagnostic (ICD-9) and Procedural (CPT) codes used to generate predictor and outcome variables.** | | | |
| --- | --- | --- | --- |
| **Variable** | **Diagnostic or Procedure Name** | **Code Type** | **Code** |
| **Reference Standard Outcome for Prediction Model of Aim 1** | | | |
| Obstructive Sleep Apnea | Obstructive sleep apnea (adult or pediatric) | ICD-9 | 327.23 |
|  | Unspecified sleep apnea | ICD-9 | 780.57 |
| Polysomno-graphy | Sleep study, simultaneous recording of ventilation, respiratory effort, ECG or heart rate, oxygen saturation, attended by a technologist | CPT | 95807 |
|  | Any age, sleep staging with 1-3 additional parameters of sleep, attended by a technologist | CPT | 95808 |
|  | Age 6 years or older, sleep staging with 4 or more additional parameters of sleep, attended by a technologist | CPT | 95810 |
|  | Age 6 years or older, sleep staging with 4 or more additional parameters of sleep, with continuous positive airway pressure therapy or bi-level ventilation, attended by a technologist | CPT | 95811 |
| **Medical Comorbidities** | | | |
| Arterial Hypertension | Malignant Essential Hypertension | ICD-9 | 401.0 |
|  | Benign essential hypertension | ICD-9 | 401.1 |
|  | Unspecified essential hypertension | ICD-9 | 401.9 |
|  | Other malignant secondary hypertension | ICD-9 | 405.09 |
|  | Other benign secondary hypertension | ICD-9 | 405.19 |
|  | Other unspecified secondary hypertension | ICD-9 | 405.99 |
| Pulmonary Hypertension | | ICD-9 | 416.0 |
| Coronary Artery Disease | Coronary atherosclerosis of unspecified type of vessel native or graft | ICD-9 | 414.00 |
|  | Coronary atherosclerosis of native coronary artery | ICD-9 | 414.01 |
|  | Coronary atherosclerosis of autologous vein bypass graft | ICD-9 | 414.02 |
|  | Coronary atherosclerosis of nonautologous biological bypass graft | ICD-9 | 414.03 |
|  | Coronary atherosclerosis of artery bypass graft | ICD-9 | 414.04 |
|  | Coronary atherosclerosis of unspecified bypass graft | ICD-9 | 414.05 |
|  | Coronary atherosclerosis of native coronary artery of transplanted heart | ICD-9 | 414.06 |
|  | Coronary atherosclerosis of bypass graft (artery) (vein) of transplanted heart | ICD-9 | 414.07 |
|  | Aneurysm of heart (wall) | ICD-9 | 414.10 |
|  | Aneurysm of coronary vessels | ICD-9 | 414.11 |
|  | Dissection of coronary artery | ICD-9 | 414.12 |
|  | Other aneurysm of heart | ICD-9 | 414.19 |
|  | Chronic total occlusion of coronary artery | ICD-9 | 414.20 |
|  | Coronary atherosclerosis due to lipid rich plaque | ICD-9 | 414.30 |
|  | Coronary atherosclerosis due to calcified coronary lesion | ICD-9 | 414.40 |
|  | Other specified forms of chronic ischemic heart disease | ICD-9 | 414.80 |
|  | Chronic ischemic heart disease unspecified | ICD-9 | 414.90 |
| Dyslipidemia | Pure hypercholesterolemia | ICD-9 | 272.0 |
|  | Pure hyperglyceridemia | ICD-9 | 272.1 |
|  | Mixed hyperlipidemia | ICD-9 | 272.2 |
|  | Hyperchylomicronemia | ICD-9 | 272.3 |
|  | Other and unspecified hyperlipidemia | ICD-9 | 272.4 |
|  | Other disorders of lipoid metabolism | ICD-9 | 272.8 |
| Ischemic Stroke | Acute ischemic stroke or cerebral infarction | ICD-9 | 434.91 |
|  | Embolic Stroke | ICD-9 | 434.11 |
|  | Thrombotic Stroke | ICD-9 | 434.01 |
| Atrial Fibrillation | | ICD-9 | 427.31 |
| **The following medical comorbidities are derived from ICD9 Codes, as defined by the Deyo Charlson Comorbidity Index**^26^**:**  Myocardial Infarction, Congestive Heart Failure, Peripheral Vascular Disease, Cerebrovascular Accident, Dementia, Chronic Pulmonary Disease, Mild Liver Disease, Moderate to Severe Liver Disease, Diabetes with Chronic Complications, Diabetes without Chronic Complications, Hemiplegia or Paraplegia, Peptic Ulcer Disease, Renal Disease, Any Malignancy including Leukemia and Lymphoma but excluding malignant neoplasm of skin, Metastatic Solid Tumor, AIDS/HIV, Rheumatic Disease | | | |
| Noninvasive Ventilation | Continuous positive airway pressure ventilation | CPT | 94660 |
|  | Respiratory Therapy | ICD-9 | 93.9 |
| **Postoperative Respiratory Complications** | | | |
| Pneumonia | Pneumococcal pneumonia [Streptococcus pneumonia] | ICD-9 | 481 |
|  | Pneumonia due to Klebsiella pneumoniae | ICD-9 | 482.0 |
|  | Pneumonia due to Pseudomonas | ICD-9 | 482.1 |
|  | Pneumonia due to Streptococcus, unspecified | ICD-9 | 482.30 |
|  | Pneumonia due to Staphylococcus, unspecified | ICD-9 | 482.40 |
|  | Pneumonia due to Staphylococcus aureus | ICD-9 | 482.41 |
|  | Methicillin resistant pneumonia due to staphylococcus aureus | ICD-9 | 482.42 |
|  | Pneumonia due to Escherichia coli [E. coli] | ICD-9 | 482.82 |
|  | Pneumonia due to other gram-negative bacteria | ICD-9 | 482.83 |
|  | Pneumonia due to other specified bacteria | ICD-9 | 482.89 |
|  | Bacterial pneumonia, unspecified | ICD-9 | 482.9 |
|  | Pneumonia, organism unspecified | ICD-9 | 486 |
|  | Pneumonia due to other specified organism | ICD-9 | 483.8 |
|  | Pneumonia in aspergillosis | ICD-9 | 484.6 |
|  | Bronchopneumonia, organism unspecified | ICD-9 | 485 |
|  | Pneumonitis due to inhalation of food or vomitus | ICD-9 | 507.0 |
| Pulmonary Edema | Pulmonary congestion and hypostasis | ICD-9 | 514 |
|  | Acute edema of lung, unspecified | ICD-9 | 518.4 |
|  | Congestive heart failure | ICD-9 | 428.0 |
|  | Fluid overload | ICD-9 | 276.6 |
|  | Other fluid overload | ICD-9 | 276.69 |
| Reintubation | Intubation, endotracheal, emergency procedure | CPT | 31500 |
|  | Ventilation assist and management, initiation of pressure or volume preset ventilators for assisted or controlled breathing; hospital inpatient/observation, initial day | CPT | 94002 |
| Respiratory Failure | Pulmonary insufficiency following trauma and surgery | ICD-9 | 518.5 |
|  | Acute respiratory failure following trauma and surgery | ICD-9 | 518.51 |
|  | Other pulmonary insufficiency, not elsewhere classified, following trauma and surgery | ICD-9 | 518.52 |
|  | Respiratory failure | ICD-9 | 518.81 |
|  | Other pulmonary insufficiency, not elsewhere classified | ICD-9 | 518.82 |
|  | Acute and chronic respiratory failure | ICD-9 | 518.84 |
