# Supplementary material for: Development and validation of a Score for Preoperative Prediction of Obstructive Sleep Apnea (SPOSA) and its perioperative outcomes
Source: BMC Anesthesiol. 2017 May 30;17:71. doi: 10.1186/s12871-017-0361-z (PMC5450400; doi:10.1186/s12871-017-0361-z)
Supplement: Supplementary file 2 — Predicted Probabilities for diagnosed Obstructive Sleep Apnea (OSA) at each integer of OSA risk prediction score. (DOCX 12 kb) [file 12871_2017_361_MOESM2_ESM.docx]

| **Table S2: Predicted Probabilities for diagnosed Obstructive Sleep Apnea (OSA) at each integer of OSA risk prediction score.** | | | |
| --- | --- | --- | --- |
| **OSA Risk Score** | **Predicted Probability** | **OSA Risk Score** | **Predicted Probability** |
| 1 | 0.0% | 26 | 2.9% |
| 2 | 0.0% | 27 | 3.5% |
| 3 | 0.0% | 28 | 4.1% |
| 4 | 0.1% | 29 | 4.9% |
| 5 | 0.1% | 30 | 5.8% |
| 6 | 0.1% | 31 | 6.8% |
| 7 | 0.1% | 32 | 8.1% |
| 8 | 0.1% | 33 | 9.4% |
| 9 | 0.1% | 34 | 11.1% |
| 10 | 0.2% | 35 | 13.0% |
| 11 | 0.2% | 36 | 15.2% |
| 12 | 0.2% | 37 | 17.6% |
| 13 | 0.3% | 38 | 20.5% |
| 14 | 0.4% | 39 | 23.6% |
| 15 | 0.4% | 40 | 27.3% |
| 16 | 0.5% | 41 | 30.8% |
| 17 | 0.6% | 42 | 34.6% |
| 18 | 0.7% | 43 | 38.7% |
| 19 | 0.9% | 44 | 43.3% |
| 20 | 1.0% | 45 | 48.2% |
| 21 | 1.2% | 46 | 52.3% |
| 22 | 1.5% | 47 | 58.5% |
| 23 | 1.7% | 48 | 61.0% |
| 24 | 2.1% | 49 | 65.6% |
| 25 | 2.5% |  |  |
