# Supplementary material for: Development and validation of a Score for Preoperative Prediction of Obstructive Sleep Apnea (SPOSA) and its perioperative outcomes
Source: BMC Anesthesiol. 2017 May 30;17:71. doi: 10.1186/s12871-017-0361-z (PMC5450400; doi:10.1186/s12871-017-0361-z)
Supplement: Supplementary file 3 — Accounting for missing data using multiple imputations by chained equations. (DOCX 14 kb) [file 12871_2017_361_MOESM3_ESM.docx]

| **Table S3. Accounting for missing data using multiple imputations by chained equations.** | | | | |
| --- | --- | --- | --- | --- |
| **Predictor** | **ORIGINAL DATASET** | | **IMPUTED DATASET** | |
|  | **OR (95% CI)** | **p-value** | **OR (95% CI)** | **p-value** |
| Male Gender | 1.24 (1.14-1.36) | <0.001 | 1.22 (1.12-1.33) | <0.001 |
| BMI (kg.m^-2^) |  |  |  |  |
| 25 to <30 | 2.13 (1.78-2.55) | <0.001 | 2.11 (1.76-2.53) | <0.001 |
| 30 to <35 | 4.04 (3.39-4.81) | <0.001 | 4.01 (3.36-4.79) | <0.001 |
| 35+ | 8.50 (7.20-10.05) | <0.001 | 8.39 (7.09-9.93) | <0.001 |
| Age (yr) |  |  |  |  |
| 18-50 | 3.56 (2.68-4.71) | <0.001 | 3.70 (2.81-4.88) | <0.001 |
| 50-70 | 2.35 (1.80-3.08) | <0.001 | 2.42 (1.86-3.15) | <0.001 |
| 70-80 | 1.55 (1.16-2.06) | 0.003 | 1.58 (1.2-2.09) | 0.001 |
| ASA |  |  |  |  |
| 2 | 3.28 (2.21-4.87) | <0.001 | 3.34 (2.28-4.9) | <0.001 |
| 3 | 3.55 (2.37-5.32) | <0.001 | 3.62 (2.45-5.34) | <0.001 |
| 4 | 2.16 (1.28-3.66) | 0.004 | 2.25 (1.36-3.7) | 0.002 |
| Arterial Hypertension | 1.67 (1.49-1.86) | <0.001 | 1.67 (1.49-1.86) | <0.001 |
| Atrial Fibrillation | 1.40 (1.21-1.61) | <0.001 | 1.40 (1.21-1.61) | <0.001 |
| Chronic Pulmonary Disease | 1.84 (1.66-2.05) | <0.001 | 1.85 (1.67-2.06) | <0.001 |
| Congestive Heart Failure | 1.35 (1.18-1.55) | <0.001 | 1.33 (1.16-1.52) | <0.001 |
| Diabetes | 1.24 (1.12-1.37) | 0.001 | 1.25 (1.13-1.38) | <0.001 |
| Dyslipidemia | 2.14 (1.93-2.37) | <0.001 | 2.20 (1.99-2.43) | <0.001 |
| Hemiplegia/Paraplegia | 1.40 (1.10-1.79) | 0.007 | 1.29 (1.02-1.65) | 0.036 |
| Liver Disease | 1.97 (1.77-2.18) | <0.001 | 2.00 (1.81-2.22) | <0.001 |
| Pulmonary Hypertension | 1.89 (1.55-2.31) | <0.001 | 1.91 (1.57-2.33) | <0.001 |
| Coronary Artery Disease | 1.20 (1.05-1.38) | 0.007 | 1.17 (1.03-1.34) | 0.020 |
| Odds ratios (OR), 95% Confidence Intervals (CI), and p-values are presented for those predictor variables identified as the strongest independent predictors in a multivariable binary logistic regression model for obstructive sleep apnea in both, the original dataset and the imputed dataset. | | | | |
